# Human Induced Pluripotent Stem Cell-Derived Cardiomyocytes for Preclinical Cardiotoxicity Screening in Cardio-Oncology

**DOI:** 10.1016/j.jaccao.2024.07.012

**Published:** 2024-09-10

**Authors:** Kyle D. Shead, Eline Huethorst, Francis Burton, Ninian N. Lang, Rachel C. Myles, Godfrey L. Smith

**Affiliations:** School of Cardiovascular and Metabolic Health, University of Glasgow, Glasgow, United Kingdom

**Keywords:** anticancer therapy, cardiotoxicity, hiPSC cardiomyocytes

## Abstract

• hiPSC-CM offer an alternative to in vivo models for predicting cardiotoxicity.
• hiPSC-CM monolayers detect pro-arrhythmic effects; inotropic detection is less established.
• Cardiac spheroids and engineered tissue may suit chronic cardiotoxicity studies (>2 weeks).
• Cardiac assays with non-myocyte cells may be key to identifying some cardiotoxicity forms.
• hiPSC-CM technologies are well placed to develop patient-specific assays in the future.

## Graphical Abstract

**Figure:**
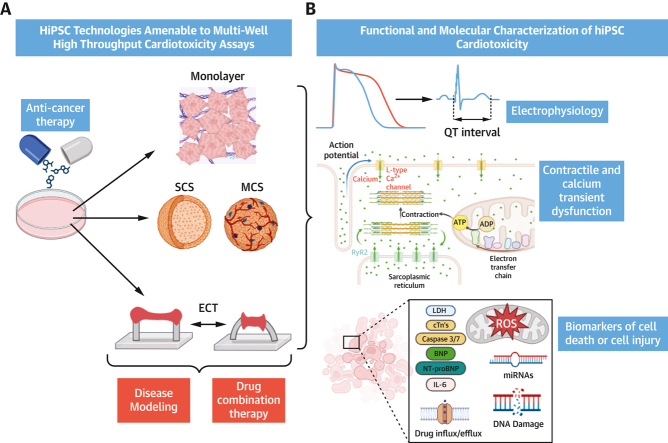

Advances in effective systemic anticancer therapies have transformed outcomes for patients with cancer. These gains have come at a cost, with cardiotoxicity evident in clinical practice. Earlier identification of cardiotoxic potential is now a major priority. Until recently, pre-clinical cardiotoxicity testing relied on in vivo rodent, canine, and non-human primate models. Limitations of in vivo testing include high cost, throughput capacity, lack of model standardization, species differences, and the 3Rs (replacement, reduction and refinement). To address these issues, there has been a shift toward the use of human induced pluripotent stem cell–derived cardiomyocytes (hiPSC-CMs) for high-throughput drug cardiotoxicity screening. hiPSC-CMs are a readily available tool for the assessment of cardiotoxic potential of anticancer drugs, through both commercial suppliers and in-house hiPSC differentiation protocols. Investigations using hiPSC-CMs allow insights into mechanisms of cardiomyocyte toxicity, pharmacogenomic interactions, and the assessment of potentially cardioprotective compounds. Furthermore, generation of patient-derived hiPSC-CMs may allow personalized, patient-specific cardiotoxicity risk prediction.^1^

Several different hiPSC-CM technologies with utility for preclinical drug screening now exist, including monolayers, 3-dimensional (3D) spheroids, and engineered cardiac tissues (ECTs). Continued development of maturation protocols is beginning to address concerns regarding the immature electrophysiology, metabolism, and structure of hiPSC-CMs compared with adult cardiomyocytes, whereby more mature hiPSC-CMs can potentially enable better prediction of cardiotoxicity in patient hearts.^2^

In this primer we focus on the 3 most promising medium- to high-throughput hiPSC-CM models for cardiotoxicity screening: 2-dimensional monolayers, cardiac spheroids, and ECTs. We compare their potential value in cardiotoxicity screening for anticancer therapies and outline potential challenges of their implementation.

## 2-Dimensional hiPSC-CM Monolayers

Monolayers consist of hiPSC-CMs seeded at relatively low densities (∼75,000-150,000 cells/cm^2^) into extracellular matrix–coated wells, forming a spontaneously beating syncytium. hiPSC-CM monolayers can be used for proarrhythmia assays using multielectrode assays or voltage-sensitive dyes.^3^ Contractile function can also be assessed by motion tracking^4^ or imaging of intracellular calcium transients using fluorescent dyes.^5^

### Advantages of 2-dimensional hiPSC-CM monolayers

hiPSC-CM monolayers are ideal for detecting acute and potentially longer term electrophysiological effects.^5^ They are routinely used to detect drug effects on cellular repolarization that underlie drug-induced QT-interval prolongation and associated increased incidence of lethal arrhythmias, specifically torsade de pointes. hiPSC-CMs have been extensively calibrated and are considered a validated in vitro electrophysiological assay by most regulatory authorities worldwide.^3^ The tyrosine kinase inhibitor vandetanib is recognized as carrying a high risk for potentially inducing torsade de pointes, validated in vitro using hiPSC-CM monolayer–based electrophysiological assays (Table 1).^3^ Assessment of hiPSC-CM monolayer contraction using image-based algorithms is noninvasive and a tested method for detecting changes in monolayer contractile dynamics in response to drugs.^4^^,^^5^ Combined with measurement of apoptotic cell markers and/or cardiomyocyte damage (eg, lactate dehydrogenase, annexin V, creatine kinase, troponins), hiPSC-CM monolayers are effective in most cardiotoxicity assay requirements.

**Table 1:** Advantages and Disadvantages of hiPSC-CM 2D Monolayers, 3D Spheroids, and ECT

Platform	Composition	Pros	Cons
2D hiPSC-CM monolayer	• hiPSC-CMs cultured on matrix-coated multiwell plate at a density of 75,000-150,000 cells/cm^2^, forming a 2D monolayer • Exhibit spontaneous synchronous contractile activity	• Medium to high throughput • Suitable for optical mapping membrane voltage • Contraction easily recorded by imaging motion • Proven platform for pro-arrhythmia assessment (CiPA)	• Quantification of contractile force not possible • Hypothesized decreased paracrine signaling between cells • Less mature hiPSC-CM phenotype • Less effective for disease modeling • Chronic studies in monolayers are challenging because of changes from steady-state contraction over longer periods
3D SCS	• ≥3,000 hiPSC-CM spherical aggregates • Formed by hanging droplet or self-assembly using low-attachment plate technologies • Spheroid diameter ranges from ∼200 μm to 1 mm	• Medium to high throughput • Increased longevity in cell culture (>24 d) • Hypothesized increased paracrine signaling between cells compared with monolayer because of increased structural contact • Improved maturation compared with 2D • Effective for disease modeling	• Diffusional limitations • Single cell type, so no paracrine signaling from other cell types • No output of contractile force • Less suited to optical mapping
3D MCS	• ≥3,000 multiple–cell type aggregates formed of hiPSC-derived and primary ECs and/or fibroblasts • Formed by hanging droplet or self-assembly	• Medium to high throughput • Increased longevity in cell culture for chronic drug exposure studies (>24 d) • Paracrine signaling from different cell types • Improved maturation • Suitable for disease modeling	• Diffusional limitations • Less suited to optical mapping • No output of contractile force • Shorter culture time than SCS because of differing proliferation rates of cell types used • Difficult to discern cell type–specific effects of therapies
3D ECT	• 250,000-500,000 hiPSC-CMs seeded in a hydrogel scaffold attached to polydimethylsiloxane pillars, forming a microtissue • Many ECTs also contain hiPSC-derived CFs and/or hiPSC-derived ECs • Cells align longitudinally, attaching to the two pillars, and contract spontaneously or with electrical stimulation	• Medium to high throughput • Increased longevity in cell culture • Direct contractile force measurement • Cells align longitudinally • Amenable to chronic drug studies • Effective for disease modeling	• Requires specialized cell culture equipment • Unknown drug absorption/adsorption to the scaffold/pillars • High cell seeding density required for cell compaction • Elevated costs

2D = 2-dimensional; 3D = 3-dimensional; CF = cardiac fibroblast; CiPA = comprehensive in vitro proarrhythmia assay; EC = endothelial cell; ECT = engineered cardiac tissue; hiPSC-CM = human induced pluripotent stem cell–derived cardiomyocyte; iPSC-CM = induced pluripotent stem cell–derived cardiomyocyte; MCS = multiculture cardiac spheroid(s); SCS = single-culture cardiac spheroid(s).

### Limitations of hiPSC-CM monolayers

Current hiPSC-CM monolayer systems have limited capacity to assess contractility because of the immaturity of the excitation-contraction coupling process. Furthermore, the nonaligned myofibrils and random attachment of cells to an unphysiologically stiff matrix (glass or plastic) makes accurate contractility monitoring technically difficult.^5^ Reduced paracrine signaling in monolayer preparations compared with intact myocardium may significantly influence maturation properties and survivability.^6^ Various techniques to enhance maturation in monolayer culture do exist,^2^ but the extent to which maturation improves the predictability of the assay has not been established, and some polymer matrices have drug absorption properties, making them problematic for cardiotoxicity screening.^5^^,^^7^

## 3-Dimensional Cardiac Spheroids

Limitations of hiPSC-CM monolayers have prompted development of 3D models for cardiotoxicity screening. Three-dimensional cardiac spheroids comprise >3,000 hiPSC-CMs that self-assemble into spheres. Single-culture cardiac spheroids (SCS) are composed solely of hiPSC-CMs, whereas multi-culture cardiac spheroids (MCS) also include cardiac fibroblasts (CFs) and endothelial cells (ECs). Multiple technologies are available for the generation of 3D spheroids, including “hanging droplet” and “ultralow surface attachment” plates, and spheroids can be scaled to suit medium- to high-throughput toxicity screening requirements in multiwell plate formats.^8^ New protocols under development include various differentiation methods and the use of matrix proteins and hydrogels. This has resulted in several diverse properties, including spheroid diameter, cell number, cellular composition, and functionality. Importantly, spheroids rely on diffusion to exchange O_2_, CO_2_, and metabolites across >100 μm of cardiac tissue to support centrally located cells, in contrast to the <10-μm distance in adult myocardium with a capillary bed. Therefore, spheroids will generate nonuniform metabolic conditions, which is an undesirable feature as the cardiotoxicity field progresses toward models closely recapitulating adult human myocardium.^9^

### Advantages of 3D cardiac spheroids

Both SCS and MCS have increased longevity and a more mature phenotype in culture compared with hiPSC-CM monolayers. This may be due to enhanced paracrine signaling, which is comparably lower in monolayer culture.^7^ Switching to 3D SCS in culture alone is enough to maintain hiPSC-CM functionality at steady state for >100 days, retaining stable electrophysiology and contractility.^9^ Similarly, in MCS preparations, hiPSC-CM survival increases upon the inclusion of CFs and/or ECs, likely further enhancing paracrine signaling among different cell types, recapitulating similar signaling networks present in whole hearts. For instance, MCS containing CFs exhibit increased expression of the mature cardiac troponin I isoform compared with SCS, which may signify more adult-like EC coupling.^6^^,^^8^^,^^9^ Paracrine signaling is an important mechanism in the cardiotoxic effects of cancer therapies, and MCS preparations can be used to elucidate specific crosstalk mechanisms. For example, doxorubicin increases the activity of endothelial nitric oxide synthase in CFs and ECs, augmenting caspase-3-driven apoptosis in hiPSC-CMs, identifying endothelial nitric oxide synthase as a target to prevent doxorubicin-related cardiotoxicity.^3^

Assessing structural changes in both cardiomyocyte ultrastructure and morphologic changes in spheroid composition elicited by toxic drug effects may have use in predicting contractile dysfunction. In this instance, changes in cell-cell structural interactions in MCS may more faithfully predict the same changes in cell interactions in patient hearts. Some preliminary data have evidenced structural contact between hiPSC-CMs and noncardiomyocyte components potentiating electromechanical coupling in MCS models. Additionally, increased perinuclear mitochondrial density, myofibril formation, limited intercalated disc formation, and the formation of simple vascular networks afford advantages in detecting structural changes incurred through specific drug treatments predisposing to negative inotropic effects.^8^^,^^10^

### Limitations of cardiac spheroids

Although image-based analysis can assay contraction in both SCS and MCS, the mechanical conditions are uncertain and variable depending on size and composition.^4^ Assays involving MCS preparations may be hindered by differential growth rates of different cell types. In particular, higher growth rate of CFs and ECs compared with hiPSC-CMs^8^ will mean that changing and non–physiologically relevant cell-type ratios may interfere with the contractile dynamics of MCS, creating difficulty in interpreting drug responses that may act by modulating growth rates of these accompanying cells.

In comparison with structurally more complex ECT models, spheroids are simpler, with little to no structural organization or cellular longitudinal alignment present in native myocardium.^6^^,^^9^ The translational relevance of toxicity resulting in distinct morphologic changes in spheroid shape and integrity is currently unexplored.

## ECTs

**Figure 1:**
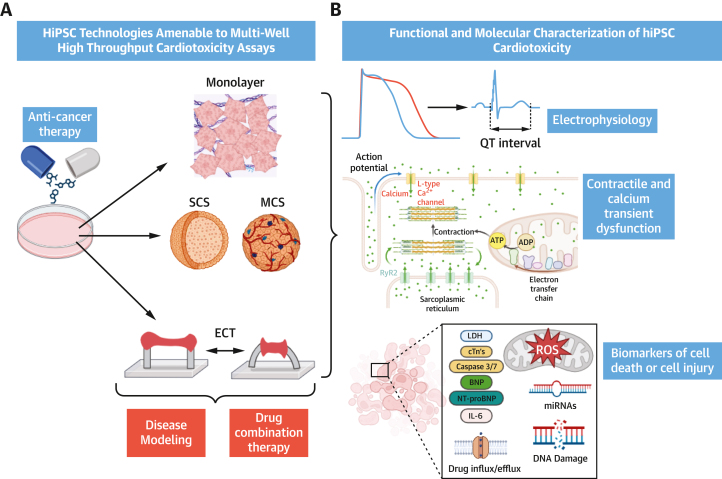
Current hiPSC-CM Technologies for Cardiotoxicity Testing (A) Human induced pluripotent stem cell–derived cardiomyocytes (hiPSC-CMs) for preclinical cardiotoxicity testing: 2-dimensional monolayers, single-culture 3-dimensional cardiac spheroids (SCS), multiculture cardiac spheroids (MCS) containing cardiac fibroblasts (CFs) and endothelial cells (ECs), and engineered cardiac tissue (ECT) (bottom). hiPSC-CMs may be used for long term drug-exposure studies of systemic anticancer therapies and further informed by disease modeling and drug combination therapies and for the assessment of cardioprotective compounds. (B) Functional and biochemical parameters to identify cardiotoxicity in hiPSC-CM models: measuring changes in membrane voltage, cell death and injury, clinically relevant biomarkers, mitochondrial function, calcium homeostasis, and contraction. ADP = adenosine diphosphate; ATP = adenosine triphosphate; BNP = brain natriuretic peptide; cTn = cardiac troponin; IL = interleukin; LDH = lactate dehydrogenase; miRNA = micro-RNA; NT-proBNP = N-terminal pro–brain natriuretic peptide; ROS = reactive oxygen species.

ECTs are formed by the seeding and compaction of >100,000 hiPSC-CMs (with or without hiPSC-CFs) to form trabeculae-like strips attached to polydimethylsiloxane pillars, which deform with ECT contraction (Figure 1).^6^^,^^7^ Different techniques, materials, and protocols can be used to generate ECTs with different composition and characteristics.^6^^,^^7^

### Advantages of ECTs

In contrast to other hiPSC-CM constructs, contractile force generation can be measured directly from ECTs and mechanical conditions (diastolic and systolic force and stiffness) monitored to allow quantification.^7^ Longitudinal alignment of hiPSC-CMs occurs in ECTs and is associated with a more mature contractile phenotype.^6^^,^^7^ These aspects represent key benefits over spheroids and monolayers, enabling the detection and quantification of reproducible drug-specific inotropic effects. Additionally, through differential culture and electrical stimulation protocols, polarized ECTs with defined atrial and ventricular sections can be generated and may allow distinction of chamber-specific toxicity.^7^

### Limitations of ECTs

A major limitation of ECT models in cardiotoxicity studies is drug absorption and adsorption to the elastomer material that constitutes the flexible pillars. This is a particular issue for hydrophobic, lipophilic-soluble compounds such as anthracyclines, tyrosine kinase inhibitors, and antibodies such as trastuzumab. This results in uncertainty in the calculation of free-drug concentrations in ECT-based assays. ECTs typically contain a minimum of about 200,000 to 500,000 cardiomyocytes per ECT, compared with 25,000 to 50,000 per assay in a 96-well plate for monolayers and spheroids. This significantly elevates costs when using both commercially sourced hiPSC-CMs or in-house differentiated hiPSCs. Standard monolayer and spheroid assays in standard 96-well plates using commercial hiPSC-CMs cost $1,000 to $2,000, compared with an equivalent throughput assay consisting of ECTs costing >$5,000.

## Future Perspectives

Wider considerations for the use of any hiPSC-CM technologies include ensuring that clinically relevant drug dosages and exposure times are recapitulated in in vitro hiPSC-CM studies. Cardiotoxicity is more likely to occur in patients with cardiovascular comorbidity or risk factors and may be modulated by other cellular stressors. As such, the use of hiPSC-CMs from healthy donors, both commercial or in house, or patient-derived hiPSC-CMs for cardiotoxicity assessment could be extended further by the development of models with modifications of the cellular environment designed to mimic those found in patients with relevant comorbidities and the “stressed” cardiovascular environment (eg, diabetes and hyperglycemia, inflammation, oxidative stress, hypertension and cardiomyocyte hypertrophy). This would aid cardiotoxicity risk prediction in subgroups of patients with cancer on the basis of comorbidities or genetic variants predisposing to cardiotoxicity. Furthermore, the potential for assessment of clinically relevant drug combinations that may potentiate cardiotoxicity is as yet unexplored, as is the use of hiPSC-CMs to evaluate the potential cardioprotective effects of coadministered drugs and compounds (Figure 1).

## Conclusions

There is an expanding role for hiPSC-CM platforms in cardiotoxicity testing. Compared with current in vivo methods, these technologies offer relatively low-cost, high-throughput capacity while removing concerns around species-specific differences. With further refinement, they should provide increasingly robust early insights into the potential cardiotoxic effects of novel anticancer agents as well as providing mechanistic clues and a platform to assess potential cardioprotective strategies, with concomitant development and implementation of patient-specific hiPSC-CMs for a personalized-medicine approach to the prevention of cancer therapy cardiotoxicity.

## Funding Support and Author Disclosures

Drs Smith, Lang, and Myles are supported by a British Heart Foundation Centre of Research Excellence grant (RE/18/6/34217). Mr Shead is supported by a British Heart Foundation 4-year PhD studentship (FS/4yPhD/F/22/34180). Dr Smith is a founder, a shareholder, and an executive of Clyde Biosciences. Dr Burton is a founder and shareholder of Clyde Biosciences. Dr Lang has received research grants from Roche Diagnostics, AstraZeneca, and Boehringer Ingelheim; and has received consultancy and speaker fees from Roche Diagnostics, Myokardia, Pharmacosmos, Akero Therapeutics, CV6 Therapeutics, Jazz Pharma, and Novartis (all outside the submitted work). All other authors have reported that they have no relationships relevant to the contents of this paper to disclose.

## References

[1] Burridge P.W., Li Y.F., Matsa E. (2016). Human induced pluripotent stem cell-derived cardiomyocytes recapitulate the predilection of breast cancer patients to doxorubicin-induced cardiotoxicity. Nat Med.

[2] Fetterman K.A., Blancard M., Lyra-Leite D.M. (2024). Independent compartmentalization of functional, metabolic, and transcriptional maturation of hiPSC-derived cardiomyocytes. Cell Rep.

[3] Blinova K., Dang Q., Millard D. (2018). International multisite study of human-induced pluripotent stem cell-derived cardiomyocytes for drug proarrhythmic potential assessment. Cell Rep.

[4] Sala L., van Meer B.J., Tertoolen L.G.J. (2018). MUSCLEMOTION: a versatile open software tool to quantify cardiomyocyte and cardiac muscle contraction in vitro and in vivo. Circ Res.

[5] Huethorst E., Mortensen P., Simitev R.D. (2022). Conventional rigid 2D substrates cause complex contractile signals in monolayers of human induced pluripotent stem cell derived cardiomyocytes. J Physiol.

[6] Zhao Y., Rafatian N., Feric N.T. (2019). A platform for generation of chamber-specific cardiac tissues and disease modeling. Cell.

[7] Mannhardt I., Breckwoldt K., Letuffe-Brenière D. (2016). Human engineered heart tissue: analysis of contractile force. Stem Cell Rep.

[8] Beauchamp P., Jackson C.B., Ozhathil L.C. (2020). 3D co-culture of hiPSC-derived cardiomyocytes with cardiac fibroblasts improves tissue-like features of cardiac spheroids. Front Mol Biosci.

[9] Fleischer S., Jahnke H.-G., Fritsche E. (2019). Comprehensive human stem cell differentiation in a 2D and 3D mode to cardiomyocytes for long-term cultivation and multiparametric monitoring on a multimodal microelectrode array setup. Biosens Bioelectron.

[10] Polonchuk L., Chabria M., Badi L. (2017). Cardiac spheroids as promising in vitro models to study the human heart microenvironment. Sci Rep.

